# Coronary artery involvement in pediatric Takayasu’s arteritis: Case report and literature review

**DOI:** 10.1186/1546-0096-11-4

**Published:** 2013-02-12

**Authors:** Shaun Mohan, Sarah Poff, Kathryn S Torok

**Affiliations:** 1 Department of Pediatrics, Children’s Hospital of Pittsburgh of UPMC, AOB Suite 5400, 4401 Penn Avenue, 15224, Pittsburgh, PA, USA; 2 Division of Pediatric Rheumatology, Children’s Hospital of Pittsburgh of UPMC, 4401 Penn Avenue, 15224, Pittsburgh, PA, USA

**Keywords:** Takayasu’s arteritis, Coronary arteries, CT angiography, Cardiac gating, ECG

## Abstract

Takayasu’s arteritis (TA) is a form of chronic vasculitis that typically occurs in young adult Asian females, but it can also present in younger patients not fitting this classic profile. In these cases, the sequelae are generally similar to those found in adults. The disease predominantly affects the aorta and its primary branches. However, the coronary arteries are also affected in up to 20% of cases, which may precipitate myocardial infarction. Imaging of the coronary arteries therefore becomes critically important in the evaluation of a patient with possible Takayasu’s arteritis. We present a case of a pediatric patient with TA who had no symptoms of angina but who was found to have significant coronary artery involvement on diagnostic imaging. This necessitated tailoring of traditional management.

## Background

Takayasu’s arteritis (TA) is a chronic vasculitis that predominantly affects the aorta, its main branches, and the pulmonary arteries. Although it is the third most common vasculitis worldwide, the cause is largely unknown [1,2]. The classic patient phenotype is an Asian woman who is between 20 to 30 years old. It is relatively uncommon in North America and Europe, with a female to male predominance of 8.5:1. In pediatrics the disease typically presents between the ages of 10 and 20 years old, but it has presented as early as 24 months of age [2]. The delay in diagnosis from initial disease manifestation can be 2–11 years in adults and sometimes longer in children [3].

In general, TA is viewed as having two phases, an acute inflammatory phase and a chronic fibrotic phase. The acute phase is characterized by a lymphocytic infiltrate with occasional giant cells of the media and fibroblast proliferation causing thickening of the intima of large vessels. Inflammation transitions to fibrosis during the chronic phase. Elastic tissue in vessel walls is replaced by collagen resulting in thickening of all three layers of the vessel. The intima may become rigid, and aneurysmal formation can occur secondary to mural stress on the vessel wall [3].

Although, the exact pathogenesis of TA is not well understood, several lines of evidence support the important role of cellular autoimmunity [4]. The vaso vasorum is thought to act as a gateway allowing the accumulation of cellular infiltrate into the vessel walls via upregulation of cellular adhesion molecules and adventitial neovascularisation [4]. The cellular infiltrate is characterized by a mixture of lymphocytes, predominately CD4+ T cells, dendritic cells, macrophages, giant cells and B cells [5]. Pro-inflammatory T-cell cytokines, such as TNF-α, IL-6 and IFN-γ, have been identified in the peripheral circulation in TA patients, both expressed in sera [6] and by peripheral blood mononuclear cells [7]. TNF-α is an important contributor to granuloma formation and has also been identified in aortic tissue of patients with TA [8], therefore implicating TNF-α as a possible therapeutic target.

Clinically, the early symptoms of the disease are nonspecific, including night sweats, anorexia, weight loss, fatigue, myalgia, and arthritis. These symptoms can be followed by unexplained hypertension. The most frequent presentation in childhood is hypertension, followed by headaches, fever, dyspnea, weight loss, and vomiting [2]. Organ manifestations arise from reduced blood supply due to vascular stenosis and subsequent ischemia. Claudication and poor or absent arterial pulses occur in areas of vessel involvement. The combination of nonspecific symptoms with decreased/absent pulses should raise suspicion for Takayasu’s arteritis, coined the ‘pulseless disease’.

The first report of coronary involvement with TA was described in 1951 [9,10]. It has since been estimated that 10-20% of all Takayasu’s patients have coronary artery involvement [3,9], based on autopsy and case series. One large cohort study of 129 patients in Korea, with a mean age of 29.5 years, found the prevalence of coronary artery involvement to be 23% (30 patients) between 1987 and 1991, when coronary arteriography was used to screen patients without symptoms of angina [11]. This series did not address the prognosis of patients with asymptomatic coronary involvement, however coronary ischemia is a recognized cause of death in these patients that warrants aggressive treatment once found [10,12]. Even if immediate ischemia does not occur due to coronary artery occlusion, vessels which have been inflamed in systemic vasculitides, such as TA, show premature atherosclerosis [12], placing patients at risk for myocardial infarction.

Three types of coronary artery lesions have been described pathologically. Type 1 is stenosis or occlusion of the coronary ostia and the proximal segment of the coronary arteries. Ostial stenosis occurs from extension of the inflammation-induced intimal proliferation and fibrous contraction from the ascending aorta and the coronary ostia [10,13]. Type 2 is diffuse or focal coronary arteritis, which can extend to all epicardial branches or may involve focal segments (skip lesions). Type 3 is the presence of coronary artery aneurysms. Type 1 is believed to be the most common lesion, while types 2 and 3 are considered uncommon [9].

Once the diagnosis of TA is made, evaluation of the extent of disease becomes important for management of its complications. To our knowledge, there is little data on the evaluation of pediatric TA patients for coronary artery involvement and even less data on the management of such patients. Because of the life threatening complications of coronary artery involvement, a thorough baseline evaluation should be performed, as the medical and surgical options for repair depend on symptoms of ischemia and grade of lesions present [10,14].

## Case presentation

A previously healthy 11 year old Caucasian boy was referred to pediatric rheumatology for recurrent febrile illnesses with anorexia, a 7 pound weight loss, and progressive fatigue for the past 11 months. His temperatures were low grade (99-100 F). He had a history of night sweats with muscle aches for a period of 4 weeks. During this time, he also developed vitiligo of the face. Further review of systems did not reveal muscle weakness, rash, joint pain, swelling, eye complaints, oral ulcers, or Raynaud’s phenomenon. Family history was unremarkable for any autoimmune or rheumatologic diseases.

Examination revealed a thin and fatigued but interactive child with mild perioral vitiligo and tachycardia. He had no bruits, but it was later noted that auscultation of the carotid pulse on the left was fainter in comparison to that on the right. He was not hypertensive and four-extremity blood pressures were not significantly different. Initial studies included a general laboratory investigation, chest X-ray, and ECG. This revealed an albumin of 3.2, total protein of 8.8, microcytic normochromic anemia, negative fecal calprotectin, elevated sedimentation rate of 91 mm/h, and C-reactive protein of 7.41 mg/dL. His IgG, IgA and IgM levels were also elevated. A chest X-ray did not reveal any cardiomegaly, masses, pulmonary infiltrates, or mediastinal adenopathy. The ECG revealed normal sinus rhythm without any evidence of ventricular hypertrophy.

**Figure 1:**
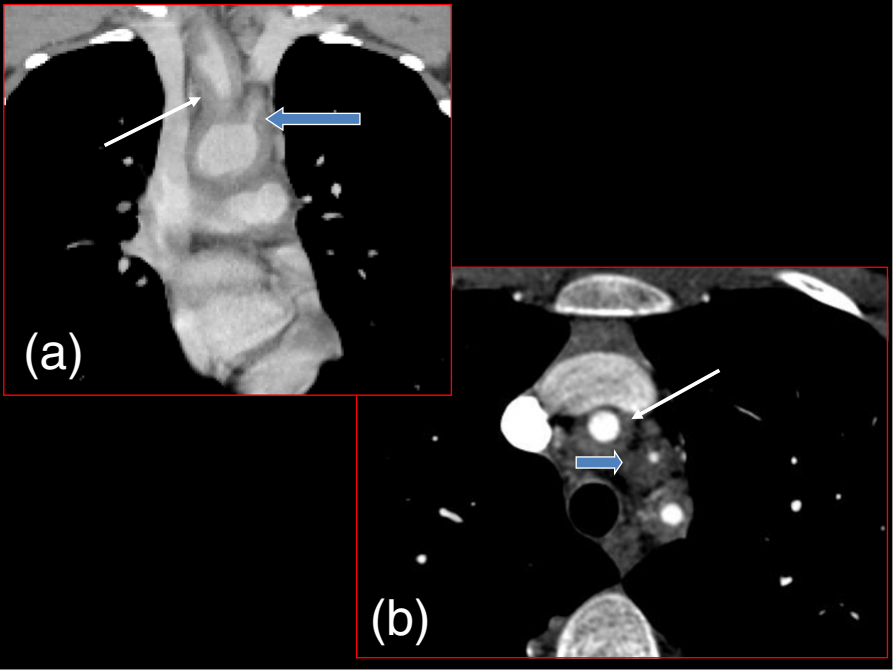
**Coronal (a) and Axial (b) CT Chest.** Concentric wall thickening at the origin of the major aortic arch branches: brachio-cephalic trunk (thin white arrow) and left common carotid artery origin (thick blue arrow).

At this point, the differential diagnosis included lymphoma, IBD, chronic deep tissue infection, occult solid organ malignancy, vasculitis, and developing connective tissue disease. Further investigation included a whole body bone scan, which did not reveal any focal uptake. A CT of his chest, abdomen, and pelvis was then obtained, and this revealed concentric thickening of the aortic arch with severe stenosis (approximately 70%) and diffuse intimal thickening of the left common carotid artery (LCCA). See Figure 1. Imaging of his abdomen did not reveal any involvement of his renal arteries or abdominal vasculature. These findings were consistent with arteritis, and given the clinical context, he was diagnosed with Takayasu’s arteritis.

Treatment was initiated with pulse doses of IV methylprednisolone while further evaluation was pursued. MRI of the brain was normal and did not support any evidence of cerebral ischemia secondary to the severe stenosis of the left common carotid artery or additional vasculitis. Cardiology was consulted to assess for cardiac ischemia, obtain a baseline echocardiogram, and provide guidance on activity restriction.

**Figure 2:**
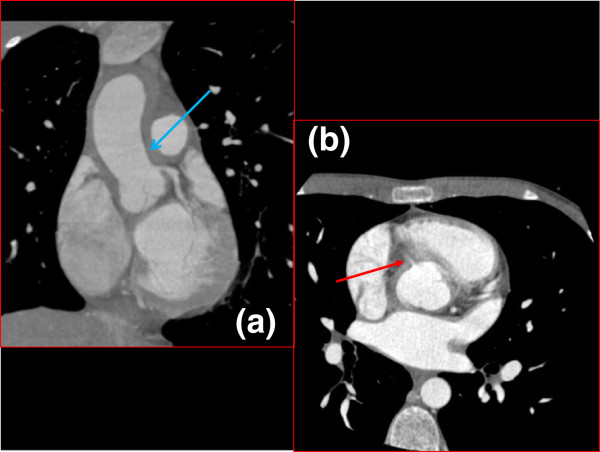
**Coronal (a) and Axial (b) Cardiac Gated CT Chest.** Fifty percent narrowing of the origin of the left coronary artery is demonstrated by arrow in (**a**, blue arrow). The right coronary artery should also be visible in this coronal section but was not due to stenosis (**a**). It can be seen as a wisp coming off the ascending aorta in the axial image (**b**, red arrow).

His echocardiogram could not visualize his coronary arteries well but did show a thickened and mildly dilated ascending aorta and normal cardiac structure and biventricular function. A CT angiogram with cardiac gating using a beta blocker was performed to better visualize the coronary arteries. See Figure 2. This revealed severe stenosis (at least 95%) of the ostium of the right coronary artery. The left coronary artery was approximately 50% stenosed and no collateral vessels were seen. Cardiac enzymes and repeat ECG did not support any cardiac ischemia. Cardiology recommended starting a daily beta-blocker to decrease tachycardia and myocardial oxygen demand, a statin to stabilize endothelium, and aspirin to prevent coagulation. These medications will be continued for the foreseeable future. Intense physical activity restriction was also recommended until a 2 month follow-up cardiac gated CT angiogram and stress treadmill test were performed.

He received 1000 mg IV solumedrol daily for 3 days. He also received IV infliximab (10 mg/kg), initially every 2 weeks and then spaced to every 4 weeks. Anti-TNF therapy was initiated because the patient was considered high risk for an MI or CVA due to the severe stenosis of his coronary and carotid arteries. We were encouraged by several case series of patients who responded well to anti-TNF therapy, especially infliximab, after failing other conventional therapies [15,16]. There were limited case reports of the use of infliximab for pediatric TA [17,18]. However, the pediatric case by N. Cakar et al. [18] did experience regression of multiple arterial stenoses, including renal, superficial femoral, and carotid arteries, after a year of infliximab treatment.

**Figure 3:**
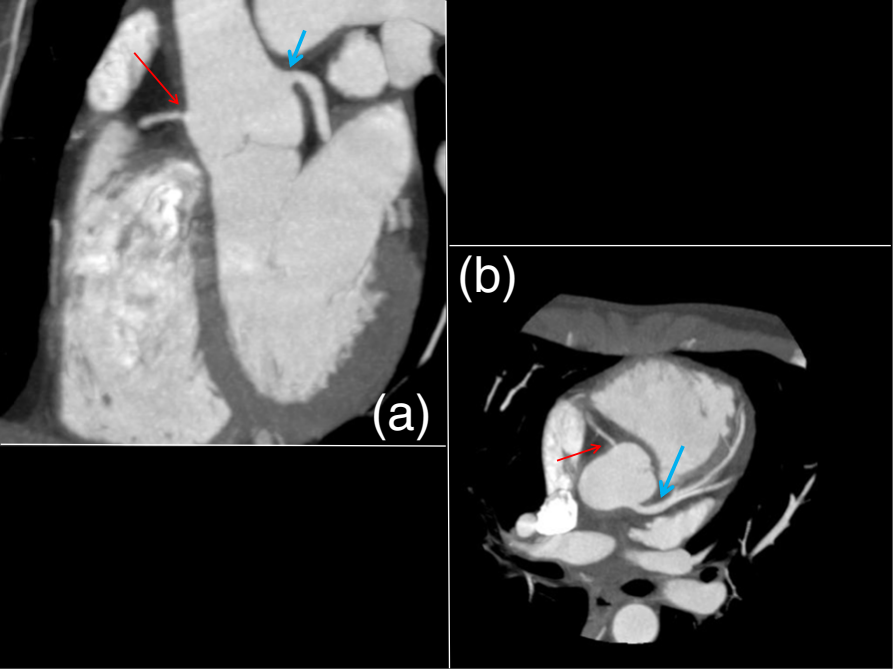
**Coronal (a) and Axial (b) Cardiac Gated CT Chest 4 months after systemic therapy.** The right coronary artery, though still stenotic, was improved and now able to be visualized on coronal section (**a**, thin red arrow). The left coronary artery opened up significantly (**b**, blue arrow).

Soon after the initiation of therapy the patient reported an increase in appetite and energy. His ESR and CRP decreased to 7 mm/hr and <0.02 mg/dl respectively after two weeks. His cardiology follow up was reassuring at two months. A stress treadmill test was performed using the Bruce Protocol for ten minutes. He achieved 97% of his maximum heart rate with no ST-T wave changes or arrhythmias. He had a normal heart rate and blood pressure response. Subsequent imaging (cardiac gated CTA and neck CTA) at 4 months from his diagnosis showed marked improvement overall. See Figure 3. There was notable reduction in the wall thickening surrounding the ascending aorta, aortic arch, and origins of the arch vessels, also the left coronary artery stenosis opened up significantly (>50%); however the right coronary artery remained severely narrowed at the ostium and the left common carotid did show persistent narrowing.

## Conclusions

Few cases of childhood onset Takayasu’s arteritis have demonstrated involvement of the coronary arteries [13,19]. Review of the literature with adult onset Takayasu’s arteritis has shown that severe coronary involvement may occur with or without symptoms of angina, as in our patient. Because these patients are at risk of serious complications, such as death from a myocardial infarction, assessment of the degree of their disease becomes paramount.

Echocardiography can yield important information with regards to cardiac function, regional wall motion abnormalities and valvar disease. While it is the initial imaging modality of choice for children to assess coronary artery anatomy in Kawasaki disease [20], it may be insufficient for older children [21], as in our patient. Patients with Kawasaki disease are typically younger and have better acoustic windows to visualize the proximal take-offs of the coronary arteries.

Due to the limitations of echocardiography, CT angiography with cardiac gating was the recommended modality for assessing our patient’s coronary arteries. Thoracic CTA with retrospective gating offered the benefits of assessing the degree of involvement in addition to providing detailed anatomy of his coronary arteries. It also facilitates motion-free evaluation of the heart, the aorta, and great vessels in addition to the coronary arteries [22]. In an adult series of patients with TA with chest pain and/or dyspnea, multi-detector CT demonstrated a high prevalence of coronary artery involvement (8 out of 18, 44%), with almost all coronary lesions being ostial or proximal in location [23]. Those patients with coronary artery involvement also had a longer disease duration compared to those without.

Other radiologic modalities to access the vasculature include conventional angiogram and MR angiogram. A conventional angiogram, which profiles the vessels in detail, is helpful when surgical intervention is necessary. However, this procedure poses additional risks by accessing large vessels, therefore it was deemed not necessary in our patient’s case. MR angiogram offers the benefit of no ionizing radiation. It is adequate and recommended for following large arch vessels in chronic vasculitidies, especially with the desire to decrease cumulative radiation exposure. For the evaluation of stenosis of smaller vessels, such as the coronary arteries, CT imaging is better. After initial echocardiogram and CT studies described, a baseline MR angiogram of the neck, chest and abdomen was performed in our patient with plans for regular MR angiogram monitoring (every 3–6 months), in addition to less frequent cardiac gated CT angiogram imaging for better coronary artery evaluation.

There is scant literature on the use of CT angiography with cardiac gating for vasculitides. Our patient presented with a rare complication of pediatric TA that required detailed imaging of his coronary arteries in order to assess his risk of complications and guide therapy and management. Thankfully, he did not show any signs of ischemia or complications thereof but this highlights an important step in the evaluation of patients with TA for guiding management.

## Consent

Informed consent was obtained from the patient and family for publication of this case report and any accompanying images.

## Abbreviations

TA: Takayasu’s arteritis;CT: Computed tomography;ECG: Electrocardiogram;MRI: Magnetic resonance imaging;RCA: Right coronary artery

## Competing interests

The authors declare that they have no competing interests.

## Authors’ contributions

All authors participated in the literature review, design, and drafting of the manuscript. In addition, all authors read and approved the final manuscript.
